# Review of and Updates on Hypertension in Obstructive Sleep Apnea

**DOI:** 10.1155/2017/1848375

**Published:** 2017-09-24

**Authors:** Masood Ahmad, Devan Makati, Sana Akbar

**Affiliations:** ^1^Department of Medicine, West Virginia University School of Medicine, Morgantown, WV, USA; ^2^Department of Medicine, Division of Nephrology, West Virginia University School of Medicine, Morgantown, WV, USA

## Abstract

Obstructive sleep apnea (OSA) is a prevalent sleep disorder as is hypertension (HTN) in the 21st century with the rising incidence of obesity. Numerous studies have shown a strong association of OSA with cardiovascular morbidity and mortality. There is overwhelming evidence supporting the relationship between OSA and hypertension (HTN). The pathophysiology of HTN in OSA is complex and dependent on various factors such as sympathetic tone, renin-angiotensin-aldosterone system, endothelial dysfunction, and altered baroreceptor reflexes. The treatment of OSA is multifactorial ranging from CPAP to oral appliances to lifestyle modifications to antihypertensive drugs. OSA and HTN both need prompt diagnosis and treatment to help address the growing cardiovascular morbidity and mortality due to these two entities.

## 1. Introduction

Obstructive sleep apnea (OSA) is a highly prevalent sleep disorder that affects 15 to 24% of all adults [1]. It is characterized by recurrent episodes of upper airway inspiratory collapse during sleep, causing breathing reduction (hypopnea) or cessation (apnea) that gives rise to transient hypoxemia and hypercapnia. Most apneic episodes are aborted by momentary arousal from sleep and a subsequent period of brief hyperventilation. This perpetual sleep fragmentation causes excessive daytime somnolence, fatigue, headaches, and decreased concentration. The severity of obstructive sleep apnea syndrome (OSAS) is classified based on the apnea-hypopnea index (number of apneic/hypopneic episodes per hour): 5–15, mild; 15–30, moderate; 30 or more, severe.

The major risk factors for OSA are obesity, male sex, and advancing age [1]. Since these conditions oftentimes predispose to and are concomitant with hypertension [2, 3] it can be confounding to determine the independent effects of OSA on the development of hypertension. However, there is increasing evidence that OSAS is independently associated with considerable cardiovascular morbidity and mortality, including ischemic heart disease, heart failure, arrhythmias, large vessel disease, and cerebrovascular disease [4–6]. There is also an increased frequency of nocturnal cardiovascular events such as angina, myocardial infarction, and sudden cardiac deaths most likely secondary to arrhythmias precipitated by nocturnal catecholamine surges [7, 8]. The acute autonomic and cardiopulmonary disturbances that are associated with repetitive nocturnal apneic episodes can lead to sustained diurnal hypertension, irrespective of other known risk factors for hypertension [9–12].

Numerous studies have shown the relation between elevated blood pressure and increased cardiovascular morbidity and mortality. However recent studies have implicated a stronger correlation of cardiovascular events with elevated ambulatory blood pressure monitor (ABPM) readings due to the elevated nocturnal readings and nondipping blood pressure patterns. Elevated nocturnal blood pressure as is noted with nocturnal catecholamine surges in patients with OSAS adds to poor outcomes. Findings from the MAPEC (Monitorizacion Ambulatoria para Prediccion de Eventos Cardiovasculares) study suggest that bedtime chronotherapy with ≥1 conventional hypertension medication to target sleep blood pressure significantly reduces cardiovascular disease risk [13]. The main acute physiological outcomes of OSAS are intermittent hypoxia, intrapleural pressure changes, and sleep fragmentation which induce endothelial dysfunction, sympathetic activation, renin-angiotensin-aldosterone system activation, and increased oxidative stress [14]. All these add to arterial stiffness and hence increased cardiovascular disease morbidity and mortality.

## 2. Prevalence

The prevalence of OSA continues to increase in developed countries in conjunction with the rise in obesity. Although its prevalence may vary across different populations and age groups, OSA has been ascertained to affect 24% to 26% of men and 17% to 28% of women between 30 and 70 years of age [15, 16]. Hypertension is also a highly prevalent disease, affecting 26.4% of all adults [17].

There is overwhelming epidemiologic evidence supporting the nexus between OSA and chronic hypertension which suggests a causal, bidirectional relationship between the two entities. Not only does OSA predispose patients to developing HTN, but also there is a greater incidence of OSA in hypertensive patients [18–20]. The prevalence of hypertension in OSA patients is estimated between 30 and 70% [21]. In patients with severe OSA, the prevalence of HTN was higher (53%) as compared to those with moderate OSA (46%) [20]. Likewise, the prevalence of OSA in hypertensive individuals is determined to be 30%–50%, which is probably an underestimate, since OSAS is markedly underdiagnosed [21–23]. The prevalence increases substantially to significant 83% if we consider just the subset of patients with resistant hypertension [24]. In fact, OSA was found to have the greatest association with resistant hypertension (64%), even more so than primary hyperaldosteronism (5.6%) [25]. Another study demonstrated a 2.5-fold elevated risk of OSA in patients with resistant hypertension relative to other hypertensives [26].

Over the years, numerous cross-sectional studies have established an association between OSA and hypertension, independently of age, weight, and other confounding factors [27–30], as a result of which OSA was listed as the most common cause of secondary hypertension in the JNC VII guidelines [31]. However, a temporal relationship between the two that implies causality is supported by two major longitudinal, prospective cohort studies, the Wisconsin Sleep Cohort Study (WSCS) [9] and the Sleep Heart Health Study (SHHS) [32]. Although both identified increased odds of developing HTN with increasing severity of OSA, the results of the SHHS that followed 2470 patients for 5 years were not statistically significant after accounting for the effect of BMI, whereas the WSCS reported a statistically significant association. This discrepancy can partly be explained by the fact that the SHHS cohort was much older than the WSCS one (average age of 60 versus 47). The strength of the association between OSAS severity and incident HTN appears to decline with age [33], a finding that is further corroborated by another study [34], where a cutoff age of 60 years was established, above which the correlation dwindles.

The WSCS found a dose-response relationship with increasing OSAS severity and incidence of HTN, independent of confounding risk factors such as age, sex, BMI, and initial BP. Those with AHI > 15 were reported to have a 3.2-fold greater odds of incident hypertension [9] and 4 times higher odds of developing nondipping nocturnal BP [35] compared to subjects with AHI < 5 at baseline. Further prospective analyses of the WSCS data suggest association of OSAS with the development of depression [36] and stroke [37]. An 18-year mortality follow-up on the WSCS sample [38] revealed that the rate of all-cause mortality was 3 times higher and the rate of cardiovascular mortality was 5.2 times higher for those with severe sleep disordered breathing (SDB), with AHI > 30 compared to those without SDB. The hazard ratio for all-cause mortality increased from 3.0 to 3.8 after excluding those who had been treated with CPAP.

Similarly, the Vitoria Sleep Cohort (VSC) [39] that observed a sample of 1180 subjects, aged 30–70 years for 7.5 years, described a positive correlation between incident hypertension and increasing SDB, but this association was attenuated and no longer statistically significant primarily after controlling for age. Adjustments for sex, BMI, neck circumference, fitness level, alcohol, tobacco, and coffee consumption further diminished the association between OSAS severity and HTN. The disparity observed among the WSCS, the SHHS, and the VSC might be attributable to the differences in the population sample and methods. The SHHS sample was older yet more obese (BMI = 28 versus 26 kg/m^2^ in VSC); more hypertensive at baseline (51% versus 28% in WSCS and 24% in VSC); and more racially diverse than the other two. The WSCS cohort was also more obese (BMI = 29) with a higher male preponderance (56% men versus 48% in the VSC) and hailed from a working population instead of the general population. While the VSC and SHHS used unattended at-home polysomnography to diagnose SDB, the WSCS employed in-laboratory polysomnography. The WSCS used an AHI of 0 as their reference point, whereas SHHS used a range of 0–4.9. The VSC used 0–2.9th quartile for Respiratory Disturbance Index (RDI) as their reference range owing to the fact that very few patients truly have an AHI of 0.

In addition, a cross-sectional study in Canada demonstrated that an increase in the AHI by one event/h was associated with a 1% risk of having hypertension [20]. Data from a prospective study in Spain [11] that examined 1,889 subjects for an average of 10.1 years also revealed an elevated incidence of hypertension in participants who did not receive CPAP therapy, compared with those who did.

Population based studies employing 24 h ambulatory BP monitoring (ABPM) have demonstrated that participants with a drop in BP of <10% during the night (nondippers) and those who mount an increase in BP at night (risers) demonstrate a greater degree of end organ damage [40], higher risk of stroke [31], incident heart failure [41], and increased risk of renal disease progression [42] as compared to those hypertensives with preserved dipping, that is, a nocturnal decrease in BP of >10% [43, 44]. Studies with ABPM in OSA patients demonstrate a higher prevalence of diminished nocturnal BP dipping compared with those without OSA [45]. A study revealed an 84% prevalence of nondipping in a sample of untreated patients with mild to severe OSA [46]. Further analysis on a subset of the WSCS cohort depicted a positive correlation between an elevated risk of nondipping and the baseline severity of OSA [35]. In addition, nocturnal CPAP use among nondippers is more effective at lowering BP than antihypertensive drugs [47], suggesting the causal role of OSA in loss of dipping.

Sex appears to influence the prevalence of OSAS, which affects males 8–10 times more commonly than females [1, 48–50] in clinical studies and approximately 2-3 times higher in epidemiological studies. The reason for the heightened male predominance remains nebulous but could allegedly be explained by factors such as fat distribution [51], upper airway anatomy [52], craniofacial configuration [53], and hormonal variation across genders [54–56]. Females with OSA tend to depict typical symptoms such as snoring, apnea, and excessive daytime sleepiness (EDS) less commonly than atypical symptoms like fatigue, depression, and anxiety [57] and have a lower AHI, generally limited to REM sleep [58]. Thus, OSA may be grossly underdiagnosed in women owing to an altered presentation [48]. Despite the high prevalence of OSAS observed in males, the effect of sex on incident HTN in OSA has been relatively inconsistent [59]. Hedner et al. [60] investigated the confounders to the association between OSA and hypertension risk and found that the odds ratio for hypertension increased across AHI tertiles from 1.0 to 2.1 (95% CI: 0.9–4.5) and from 1.0 to 3.7 (95% CI: 1.7–8.2) in males but not in females where the OR increased from 1.0 to 1.8 (95% CI: 0.8–3.9) and 1.0 to 1.6 (95% CI: 0.7–3.5).

## 3. Pathophysiology of HTN in OSA

**Figure 1:**
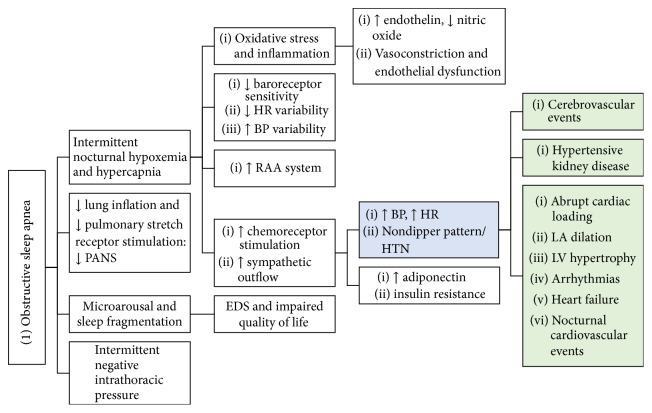
Mechanism of hypertension and end organ damage in obstructive sleep apnea. PANS, parasympathetic autonomic nervous system; RAA, renin-angiotensin-aldosterone; EDS, excessive daytime sleepiness; LV, left ventricular; LA, left atrial.

The pathophysiology of hypertension in OSA is complex and is dependent on various factors such as sympathetic tone, peripheral vasoconstriction, increased renin-angiotensin-aldosterone activity, and altered baroreceptor reflexes (Figure 1). The intermittent apneic episodes cause hypoxemia, which stimulates the carotid body chemoreceptors, causing reflex sympathetic stimulation of the medullary cardiorespiratory centers. The nocturnal catecholamine surges cause a resultant nocturnal increase in heart rate and blood pressure that is most prominent during the postapneic hyperventilation, soaring as high as 240/130 mmHg [61, 62]. The nocturnal BP surge manifests in many as failure of the normally observed “dipping” phenomenon [63] and in other cases precipitates cardiovascular events such as coronary spasm, angina, and arrhythmias.

Tamisier et al. [64] in their study found that intermittent hypoxia significantly increased daytime ambulatory blood pressure after a single night exposure (3 mm for mean diastolic) and further increased daytime pressures after 2 weeks of exposure (8 mm Hg systolic and 5 mm Hg diastolic) with no evident changes in either vascular reactivity or systemic inflammatory markers. The study also assessed muscle sympathetic nerve activity (MSNA) and found that MSNA increased across exposure also while baroreflex control of sympathetic outflow declined, thus suggesting that sympathoactivation induced by intermittent hypoxia likely contributes to blood pressure elevation and may derive from reduced baroreflex inhibition.

In addition to the increased sympathetic response to hypoxia, there is also a simultaneous reflex stimulation of the respiratory centers causing increased depth and frequency of breathing. Lung inflation reflexively stimulates the vagal stretch receptors, mitigating the increased sympathetic activity, hence maintaining autonomic homeostasis [65–67]. In the absence of adequate lung inflation during apneic episodes, sympathetic outflow remains unchecked, resulting in an exaggerated sympathetic response to hypoxemia. This amplified chemoreflex sensitivity causes a baseline elevated sympathetic tone in OSA, which persists during the day even in the absence of hypoxia [68, 69]. In addition to impaired pulmonary stretch receptor and baroreceptor reflexes, blunted heart rate variability and increased BP variability are observed in OSAS [70], which is indicative of underlying autonomic dysfunction [71] and is a predictive marker for developing hypertension [72] and potential cardiovascular disease [73]. It is also associated with an increased risk of end organ damage in hypertensive individuals [74] and adverse outcomes in patients with preexisting cardiovascular disease [75].

OSAS causes intermittent negative intrathoracic pressure, which, coupled with transient nocturnal catecholamine surges, exerts profound mechanical stress on the heart, gradually causing left ventricular hypertrophy and atrial remodeling, thereby increasing the risk of heart failure and arrhythmias such as atrial fibrillation [76], even in the absence of sustained diurnal hypertension, as evaluated by 24 h ABPM.

Decreased sympathetic and increased parasympathetic activity during NREM sleep, which comprises the majority of sleep time, contributes to the normal circadian variation of blood pressure, causing “dipping” of both systolic and diastolic BP at night, approximately by 10–15% [77, 78]. NREM is intermittently punctuated by REM sleep, characterized by predominant sympathetic activity and transient nocturnal BP surges. The generalized skeletal muscle atonia in REM sleep predisposes the already susceptible airway in OSAS patients to collapse, exacerbating apneic episodes and further intensifying the nocturnal sympathetic hyperactivity.

Recurrent nocturnal hypoxemia with subsequent reoxygenation increases oxidative stress to the body, much like ischemia reperfusion injury, inducing release of reactive oxygen species, inflammatory cytokines, and vasoactive substances, thereby mediating endothelial damage [79–82]. OSAS patients demonstrated attenuated small vessel response to vasodilators such as acetylcholine in some studies [79, 83]. Carlson et al. [83] demonstrated in their study that forearm blood pressure flow after acetylcholine was reduced in OSA patients compared with that in controls, but there was no difference between hypertensive and normotensive subjects. However, this finding was not consistent with other studies [84]. OSAS patients were also found to have reduced levels of the vasodilator nitrogen monoxide, where the degree of decrease in levels is consistent with the severity of OSAS [85] and is also responsive to CPAP therapy [86].

Endothelin levels have been shown to increase in hypoxia in in vitro studies [87]. OSAS patients were found to have high nocturnal levels of endothelin-1 compared to controls [88], the magnitude of which corresponds to the degree of BP elevation and increasing AHI [89]. Furthermore, elevated endothelin levels were shown to decline following treatment with CPAP for 4 hours, implicating the role of endothelin in the pathogenesis of HTN in OSA patients [88, 89]. Elevated levels of C-reactive protein have also been observed in OSAS patients. Those with impaired nocturnal BP dipping were reported to have higher levels of CRP compared to those with relatively preserved dipping [90]. In addition, sleep deprivation can independently induce systemic inflammation [91, 92]. The synergistic combination of recurrent hypoxemia and sleep deprivation in OSAS patients may cause increased production of inflammatory mediators, upregulation of leucocyte adhesion molecules [93–95], serum amyloid A [96], CRP [97, 98], circulating angiogenic inhibitors [99, 100], and decreased cGMP [101], predisposing OSAS patients to endothelial injury and dysfunction, vasoconstriction, and subsequent vascular events, Figure 1.

Obesity is rampant in OSAS patients and is a well-known independent risk factor for glucose intolerance. Other mechanisms responsible for insulin resistance include enhanced resting sympathetic tone causing reduced levels of adiponectin [102], recurrent hypoxemia, and cumulative sleep debt. Several [103, 104] studies have endorsed an association of OSA with insulin resistance independent of obesity [105], which is further supported by trials that demonstrate mild reversal of glucose intolerance with CPAP use in OSA patients [106]. Seetho et al. [107] reported that patients with OSA with obesity have increased arterial stiffness that potentially influences cardiovascular risk independently of metabolic abnormalities. Arterial stiffness correlated with mean arterial blood pressure (*P* = 0.003) and obstructive sleep apnea severity (AHI; *P* < 0.001).

Another mechanism which potentially contributes to HTN in OSAS is increased activity of the renin-angiotensin-aldosterone system (RAAS). Periodic hypoxia has been shown to boost angiotensin I expression and angiotensin II receptor stimulation in the carotid body [108, 109] as well as increasing renin and aldosterone levels in animal studies [105–108]. A meta-analysis conducted on 13 studies observed increased Ang II levels in OSAS patients relative to controls, while elevated aldosterone levels were seen in OSAS patients with HTN compared to normotensive OSAS patients [110]. These elevated markers were seen to decline following CPAP therapy and subsequent BP reduction [111–114], thereby supporting the causal role of RAAS activation in OSA-mediated hypertension. Conversely, OSA was also found to be more prevalent in patients with hyperaldosteronism than those without (18% versus 8.8%), with hyperaldosteronism patients being 1.8 times more likely to have OSA after adjusting for confounding risk factors [115]. Excess aldosterone causes fluid retention, which, coupled with rostral fluid displacement during sleep, promotes accumulation of fluid within the neck, hence causing increased upper airway obstruction [115–127]. The fact that spironolactone helps reduce OSA severity by 50% in patients with resistant hypertension further testifies to the potential contribution of aldosterone in developing or exacerbating preexisting OSAS [128].

The nexus between OSA and HTN appears to be bidirectional according to epidemiological studies [18, 129]. The idea of reciprocal causality has been supported by experimental data in animals where acute BP surges have been shown to increase upper airway obstruction [130–132]. Accordingly, similar results have been demonstrated in humans where phenylephrine mediated rise in BP resulted in lower daytime genioglossus electromyographic activity [133]. Potential mechanisms that can contribute to upper airway collapsibility include inhibitory effect of baroreceptor activation on upper airway dilatory muscle or a change in brain perfusion by upsurge in mean arterial pressure [134].

## 4. Treatment

### 4.1. CPAP Therapy

While CPAP remains the mainstay therapy for OSAS, its effect on blood pressure reduction has been variable, most likely owing to the multifactorial nature of hypertension. CPAP therapy has been observed to attenuate the nocturnal sympathetic surge and mediate acute reduction in nocturnal BP in OSAS patients [68, 135–138]. However, results of several trials that evaluated the impact of long-term CPAP therapy on daytime blood pressure reduction in OSAS patients have been unremarkable [10, 139]. Numerous meta-analyses [10, 140–147] have demonstrated only a mild decrease in BP of about 1.3 to 3 mm Hg with CPAP. This modest BP lowering effect of CPAP, although not comparable to antihypertensive drugs, is significant nonetheless in improving cardiovascular and cerebrovascular sequelae by decreasing mortality by 6%–8% for stroke and 4%-5% for ischemic heart disease [148, 149].

The generalizability of the results of these trials however is limited owing to variability in factors affecting both the study population (such as age, sex, BMI, baseline severity of both hypertension and OSA, and concomitant use of antihypertensive drugs) and methodology (such as sample size, method of BP evaluation, e.g., office BP versus ABPM, use of placebo for control group, CPAP compliance, duration of CPAP used per night, and overall length of treatment and follow-up). The magnitude of the drop in BP is subject to various factors such as CPAP compliance [145, 146, 150, 151], duration of treatment with CPAP [145, 152] and its use during REM sleep [153], presence of EDS [146, 150], baseline BP [145, 147], and severity of OSAS with increased benefits seen in patients with baseline AHI > 30 [140, 146] and a high BMI [141, 154]. Patients who exhibited higher baseline BP, untreated hypertension, nocturnal hypertension/nondipper pattern, and resistant hypertension were found to benefit the most with CPAP therapy [141, 155–157]. CPAP therapy was observed to have a greater antihypertensive effect in patients with resistant hypertension than in those without [151, 156, 158–161], with a drop of −7.21 (95% CI, −9.04 to −5.35; *P* < 0.001; *I*^2^, 58%) and −4.99 (95% CI, −6.01 to −3.96; *P* < 0.001; *I*^2^, 31%) observed in SBP and DBP, respectively, according to a meta-analysis [162]. In nondippers, consistent use of CPAP led to the recovery of the nocturnal dipping pattern [156] which helps improve cardiovascular morbidity and mortality since those with nocturnal rising BP pattern have the greatest cardiovascular risk [163, 164]. These findings were corroborated by the HIPARCO Randomized Clinical Trial [151] conducted in Spain, which showed higher dipping patterns in patients who received CPAP therapy compared to those who did not (35.9% dipping in CPAP group versus 21.6% in control; adjusted OR = 2.4; *P* = 0.02). The BP lowering effect was also more pronounced in patients who used CPAP for at least 4 hours per night, with a drop of 1.3 mm Hg in BP detected per hour of CPAP therapy used.

A randomized controlled trial [152] was conducted, comparing the effect of therapeutic and subtherapeutic CPAP in patients with moderate to severe OSA. In the therapeutic CPAP group, treatment pressure was increased until apneas, hypopneas, and snoring were prevented during all sleep stages with the patient lying supine. Mean effective treatment pressure was 9.1 ± 2.3 cm H_2_O (between 6 to 12 cm H_2_O). In the subtherapeutic treatment group, pressure was left unchanged at the lowest possible value for the CPAP device used (3 or 4 cm H_2_O). After an average treatment of 9 weeks, far substantial reductions in mean BP (9.9 ± 11.4 mm Hg) and both nocturnal and daytime systolic and diastolic BP (10 mm Hg approximately) were noted in the therapeutic CPAP group, most likely owing to the length of the trial and treatment pressure used (compared to 1.3–3 mm Hg in most trials lasting for about 4–6 weeks). The fact that, despite causing 50% reduction in AHI, subtherapeutic CPAP was not effective enough to cause any relevant change in BP (*P* = 0.01) further highlights the significance of highly effective therapy. Since OSA-mediated hypertension has a chronic etiology involving endothelial dysfunction and cardiovascular remodeling, it is very possible that short-term controlled studies may not entirely reveal the actual potentially far reaching consequences of scrupulous long-term CPAP therapy on hypertension and its cardiovascular sequelae.

Notwithstanding increased benefits of CPAP therapy in those patients who demonstrated more than 50% reduction in AHI following CPAP [152], this effect seems independent of normalization of oxygen saturation alone. A trial comparing CPAP versus oxygen therapy in OSAS patients demonstrated BP reductions in the CPAP group but not in the oxygen group, which, despite correcting the nocturnal hypoxia, did not lower BP [165].

CPAP mediated BP reduction is also contingent on the presence of daytime somnolence, with far lower efficacy observed in patients who did not report EDS, despite having severe OSA with AHI > 30/h [154, 166, 167]. Even those patients who experienced only modest subjective somnolence also reported some improvement in BP with CPAP therapy [168]. Additionally, those with fewer daytime symptoms are less likely to adhere to CPAP treatment, hence eliciting fewer benefits.

### 4.2. Lifestyle Modifications

Since obesity is the single most important risk factor for OSA, even modest reductions in weight help attenuate the severity of both OSA and OSA induced hypertension. In a study [169] that examined a sample of participants from the Wisconsin Sleep Cohort Study (WSCS) a 10% increase in weight predicted a 32% increase in the AHI and a 6-fold increase in the odds of developing moderate to severe SDB. Conversely, a 10% weight loss predicted a 26% decrease in the AHI.

Since both OSA and obesity have an independent causal relationship with hypertension, an integrated approach that includes lifestyle modifications such as weight loss should be recommended in OSA patients receiving treatment with CPAP and antihypertensives. In a randomized control trial [170] assessing the impact of weight loss and CPAP on OSA, patients were assigned to receive treatment with CPAP alone, a weight-loss intervention, or combined CPAP and weight-loss intervention for 24 weeks. A greater reduction was observed in the combined-intervention group (14.1 mm Hg) than in either the weight-loss group (6.8 mm Hg) or the CPAP group (3.0 mm Hg). Combination therapy was also associated with a significantly greater drop in mean arterial pressure. These findings suggest the possibility of a synergistic interaction effect between lifestyle modifications and weight-loss and CPAP therapy in the management of hypertension in OSA patients.

### 4.3. Oral Appliances

Oral appliances are a recommended alternative treatment to CPAP in patients with mild to moderate OSA. There have been only limited clinical studies on this intervention, most of which were observational with small sample sizes and short durations. A meta-analysis of seven studies involving 399 OSAS patients [171] found a beneficial effect of oral appliances on BP comparable to that of CPAP. The average drop in SBP, DBP, and MAP was reported to be −2.7 mm Hg (95% CI: 0.8 to 4.6; *P* = 0.04), −2.7 mm Hg (95% CI: 0.9 to 4.6; *P* = 0.004), and −2.40 mm Hg (95% CI: 4.01 to 0.80; *P* = 0.003), respectively. Since most of the studies in this meta-analysis were also observational, the true effect of oral appliances on BP reduction in OSA remains nebulous. Andren et al. [172] in their study found that, in patients with OSA and hypertension, oral appliances therapy had a modest trend towards effect on reducing blood pressure. The trend was stronger towards treatment effect when patients with normal baseline ambulatory blood pressure were excluded. As per the systemic review by Okuno et al. [173] although oral appliances have demonstrated good efficacy in patients with mild to severe levels of OSA, they are not completely effective in all patients. Studies vary in the degree of bias and the definitions applied for treatment success. More recent data suggests that oral appliance therapy should be prescribed to patients with OSA who are intolerant to CPAP therapy or prefer alternate therapy as oral appliances are noninferior to CPAP on blood pressure and have better compliance and lastly CPAP and oral appliances lowered morning blood pressure [174].

### 4.4. Upper Airway Surgery

Surgical options such as tonsillectomy and uvulopalatopharyngoplasty (UPPP) have also been looked at for impact of blood pressure in patients with OSAS. The SKUP3 randomized controlled trial by Di Munro et al. [175] showed that modified UPPP significantly improved sleepiness, nocturnal respirations, and quality of life as well as lowering blood pressure significantly after surgery in a selected group of patients with moderate to severe OSA. Tonsillectomy and adenoidectomy are surgeries used to treat OSA in children. Gaddam et al.  [128] from their care series inferred that, among children receiving tonsillectomy and adenoidectomy for OSA treatment, nonobese children improved more than obese children did in terms of blood pressure with a significantly decreased nocturnal DBP index (−12.0 to 18.8, *P* = 0.18) and morning SBP (111.1 to 105.8 mm Hg; *P* = 0.014), SBP index (−5.4 to −10.9, *P* = 0.008), and DBP (−12.0 to −18.7, *P* = 0.023).

### 4.5. Antihypertensive Drugs

Hypertensive patients with mild to moderate OSA who do not need CPAP are ideal candidates for hypertensive therapy, as are those with severe OSA who do not tolerate or are not compliant with CPAP. Due to lack of adequate evidence, there are no specific guidelines as to which class of antihypertensive medications should be used to treat hypertension in OSA patients. Hypothetically, however, owing to the pathophysiological mechanisms causing and maintaining HTN in OSA such as the sympathetic and RAAS overactivity, antihypertensive drugs that modulate the activity of these systems such as B-blockers and aldosterone antagonists may be the best treatment options for hypertension in OSA patients. Aldosterone levels are generally normal in OSA except in patients with treatment resistant hypertension or severe OSA [175]. The aldosterone antagonist spironolactone has been very effective in decreasing the severity of OSA [128]. ACE inhibitors, angiotensin receptor blockers, and aldosterone antagonists have a moderate antihypertensive effect in moderate OSA. In severe OSA, aldosterone antagonists may be even more effective [176]. In a study [177] comparing B-blockers and calcium channel blockers, 31 patients were treated for 6 weeks with either nebivolol (*n* = 16) or valsartan (*n* = 15). While both the drugs effectively reduced both systolic and diastolic BP, nebivolol had a significantly more marked impact on the decrease of heart rate compared to valsartan (*P* < 0.001), which may help in patients with nocturnal tachycardia. In a study by Kraiczi et al. [178], B-blockers (atenolol) significantly reduced nocturnal diastolic and systolic BP more effectively compared to calcium channel blockers, ACE inhibitors, and angiotensin receptor blockers (but not hydrochlorothiazide). However, there was no difference noted in daytime BP and OSA severity across the different classes of antihypertensives.

According to most studies, antihypertensive drug mediated nocturnal blood pressure reduction has not been found to be associated with any significant decrease in the severity of sleep disordered breathing as measured by AHI, suggesting nocturnal high BP contributes only minimally to apneic events and OSA severity. The results have been observed across all the commonly used classes of antihypertensive drugs including B-blockers, calcium channel blockers, angiotensin-converting enzyme (ACE) inhibitors, angiotensin receptor blockers (ARBs), hydrochlorothiazide, methyldopa, and clonidine [178–181]. However, one study [134] revealed a weak correlation between the influence of drug therapy on reduction of nocturnal BP and frequency of apneic episodes during REM sleep in hypertensive OSA patients despite no change in AHI during NREM sleep or for the overall sleep duration. This further supports the fact that any potential effect of elevated BP on upper airway collapse may be sleep stage dependent. Data from a few studies also demonstrated some resolution of apnea/hypopnea following antihypertensive drug therapy. Studies comparing B-blockers and ACE inhibitors in hypertensive OSA patients [182–184] revealed that both Metoprolol and cilazapril caused a statistically significant decrease in both nocturnal BP and AHI. However, according to one of the studies the effect of cilazapril was observed in all sleep phases and it was hence more favorable for the sleep disturbance pattern characteristic to OSA as compared to Metoprolol, which did not cause any changes during REM sleep [182]. Diuretics, particularly spironolactone, have been shown to be the most promising medication that relieves pharyngeal edema and secondary upper airway destabilization, hence improving both OSA severity and HTN [119, 128].

However, most of these studies had extremely limited samples (12–24 patients), variable populations, short duration of treatment and follow-ups, and lack of placebo effect and used noninvasive methods to measure BP. Since BP varies greatly during apneic cycles (between 150 and 300 mm Hg), invasive BP measurement is the most accurate technique to evaluate the mean nocturnal BP in patients with sleep disordered breathing. Hence there exists a lack of definitive recommendations regarding the use of antihypertensive agents for OSA treatment.

Conversely, there has been some incriminating evidence advising against the use of ACE inhibitors in hypertensive OSA patients. According to Cicolin et al. [185], enalapril induced a dry cough and increased upper airway inflammation (measured by exhaled nitric oxide, a marker of airway inflammation) subsequently causing OSA exacerbation, all of which resolved following cessation of enalapril. B-blockers have been shown to cause increased weight, which can aggravate OSA [185]. In addition, study on 186 OSA patients with HTN [186] reported significant sleep impairment associated with the use of calcium channel blockers with a reduction of 41 minutes in total sleep time (*P* = 0.005) and an 8% decrease in sleep efficiency (*P* = 0.004). On the other hand, no other antihypertensive drug, such as diuretics and beta-blockers, was found to have an adverse impact on sleep duration. Notwithstanding these results, it is worth mentioning that most of these adverse effects need a long time before they can be discernable. Hence, the limited duration of these studies is not adequate to assess the true impact that these medications have on OSA severity. Walia et al. [187] analyzed the data from the HeartBEAT (Heart Biomarker Evaluation in Apnea Treatment) trial and found that in patients at high cardiovascular risk or established cardiovascular disease there was a strong association of severe untreated OSA and resistant elevated blood pressure despite treatment with an aggressive antihypertensive medication regimen. Thus in these patients a combination of antihypertensive medications and other strategies such as CPAP should be used.

## 5. Conclusion

There are numerous mechanisms that can potentiate the development of hypertension in OSA, with wide variation in individual susceptibility. These include increased sympathetic tone, inflammation, endothelial dysfunction, peripheral vasoconstriction, increased RAAS, heightened chemoreflex, and blunted baroreflex sensitivity. Conversely, acute rise in BP may cause an inhibition of the upper airway muscles. This, coupled with volume overload due to increased activity of the RAAS and rostral fluid displacement during sleep, can cause pharyngeal edema and subsequent airway obstruction, thus causing OSA in hypertensive patients. Treatment options for OSA range from surgical options such as tonsillectomy and adenoidectomy in children to modified UPPP in adults, to noninvasive measures such as CPAP therapy, oral appliances, and lifestyle modifications to address obesity; and lastly use of antihypertensive drugs has been investigated. These measures help address the issues of resistant hypertension that are frequently found in patients with OSAS which is linked with cardiovascular morbidity and mortality. Hence OSA and HTN both need prompt diagnosis and treatment to help address the growing cardiovascular morbidity and mortality due to these two entities. Continued advancements in the field of medicine may lead to newer treatment modalities in the future.
